# Strange Superstition

**Published:** 1854-07

**Authors:** 


### STRANGE SUPERSTITION.

The Norwich (Conn.) Courier relates a strange and almost incredible tale of superstition recently enacted at Jewett City, in that vicinity. About six years ago, Horace Ray, of Griswold, died of consumption. Since that time, two of his children, grown up people, have died of the same disease, the last one dying some two years since. Not long ago the same fatal disease seized upon another son, whereupon it was determined to exhume the bodies of the two brothers already dead, and *burn them, because the dead were supposed to feed upon the living;* and so long as the dead body in the grave remained in a state of decomposition, either wholly or in part, the surviving members of the family must continue to furnish the sustenance on which that dead body fed. Acting under the influence of this strange and blind superstition, the family and friends of the deceased proceeded to the burial ground at Jewett City on the 8th inst., dug up the bodies of the deceased brothers, and burned them on the spot. It seems impossible to believe that such dark ignorance and folly could exist in the middle of the nineteenth century, and in a state calling itself enlightened and Christian.—*Boston Courier.*

